# An unusual presentation of non-specific cystic degeneration of craniofacial fibrous dysplasia: a case report and review of literature

**DOI:** 10.1186/s40902-020-00275-2

**Published:** 2020-09-16

**Authors:** Inseok Hong, Dong Cheol Kang, Dae-Ho Leem, Jin-A Baek, Seung-O Ko

**Affiliations:** 1 Department of Oral and Maxillofacial Surgery, School of Dentistry, Chonbuk National University Dental Hospital, 20, Geonji-ro, Deokjin-gu, Jeonju-si, Jeollabuk-do Republic of Korea; 2 Research Institute of Clinical Medicine-Biomedical Research Institute, Chonbuk National University Hospital, 20, Geonji-ro, Deokjin-gu, Jeonju-si, Jeollabuk-do Republic of Korea

**Keywords:** Craniofacial fibrous dysplasia, Cystic degeneration, Cyst enucleation, Fibrous dysplasia, Mandible, Maxilla, Polyostotic fibrous dysplasia

## Abstract

**Background:**

Fibrous dysplasia (FD) is a rare, sporadic, and benign congenital condition in which normal cancellous bone is replaced by fibro-osseous tissue with immature osteogenesis. FD localized in the cranial and facial bones is called craniofacial fibrous dysplasia (CFD). Cystic degeneration in CFD cases is rare; cystic degeneration appearing in both the maxilla and the mandible FD lesion is even rarer. The aim of this article was to report a case of fibrous dysplasia of the mandible and maxilla complicated by nonspecific cystic degeneration.

**Case presentation:**

A 30-year-old woman presented with a rare case of non-specific cystic degeneration in a mandible and maxilla FD lesion that occurred 11 years after surgery. She was diagnosed with polyostotic CFD and underwent maxillary and mandibular bone contouring. Cyst enucleation under general anesthesia was performed in the mandibular region due to pain and discomfort.

**Conclusions:**

In cases involving non-aggressive and non-invasive FD cystic degeneration in focal areas, conservative treatment is recommended. However, if cystic degeneration of FD develops rapidly and causes discomfort, pain, or dysfunction, surgical treatment should be considered.

## Background

Fibrous dysplasia (FD) is a benign disorder characterized by the replacement of normal bone tissue with proliferative fibrous connective tissues [1]. Somatic mutations in the Gs-alpha gene on chromosome 20 can lead to endocrine tumors, FD, and McCune–Albright syndrome (MAS) [2]. FD occurs in two forms: the monostotic form affecting one bone (approximately 70% of cases), and the polyostotic form affecting at least two bones (approximately 30% of cases) [3]. FD that appears in the cranial and facial bones is called craniofacial fibrous dysplasia (CFD). The prevalence of polyostotic and monostotic CFD is 71–91% and 10–29%, respectively [4, 5]. In the jaw bone, FD is approximately twofold more prevalent in the maxilla and usually occurs unilaterally [1].

Non-epithelial-lined cysts sometimes occur in association with various benign and malignant bone lesions, including FD, giant cell tumors, chondroblastoma, ossifying fibroma, benign osteoblastoma, cemento-osseous dysplasia, fibrous histiocytoma, fibrosarcoma, and osteosarcoma. These cysts show various patterns and can appear as aneurysmal bone cysts, simple bone cysts, or non-specific cystic degenerations [6].

Cystic degeneration in CFD cases is rare; cystic degeneration appearing in both the maxilla and the mandible is even rarer. A PubMed search from 1946–2019 (using the search terms “fibrous dysplasia” [Ti] OR “McCune–Albright” [Ti] OR “Jaffe–Lichtenstein” [Ti] OR “Mazabraud” [Ti] AND (cyst [Ti] OR cystic [Ti]) initially identified 78 articles. After screening and manual review, a total of seven articles on the occurrence of cystic degeneration in maxillary or mandibular FD were identified [6–12].

## Case presentation

**Fig. 1:**
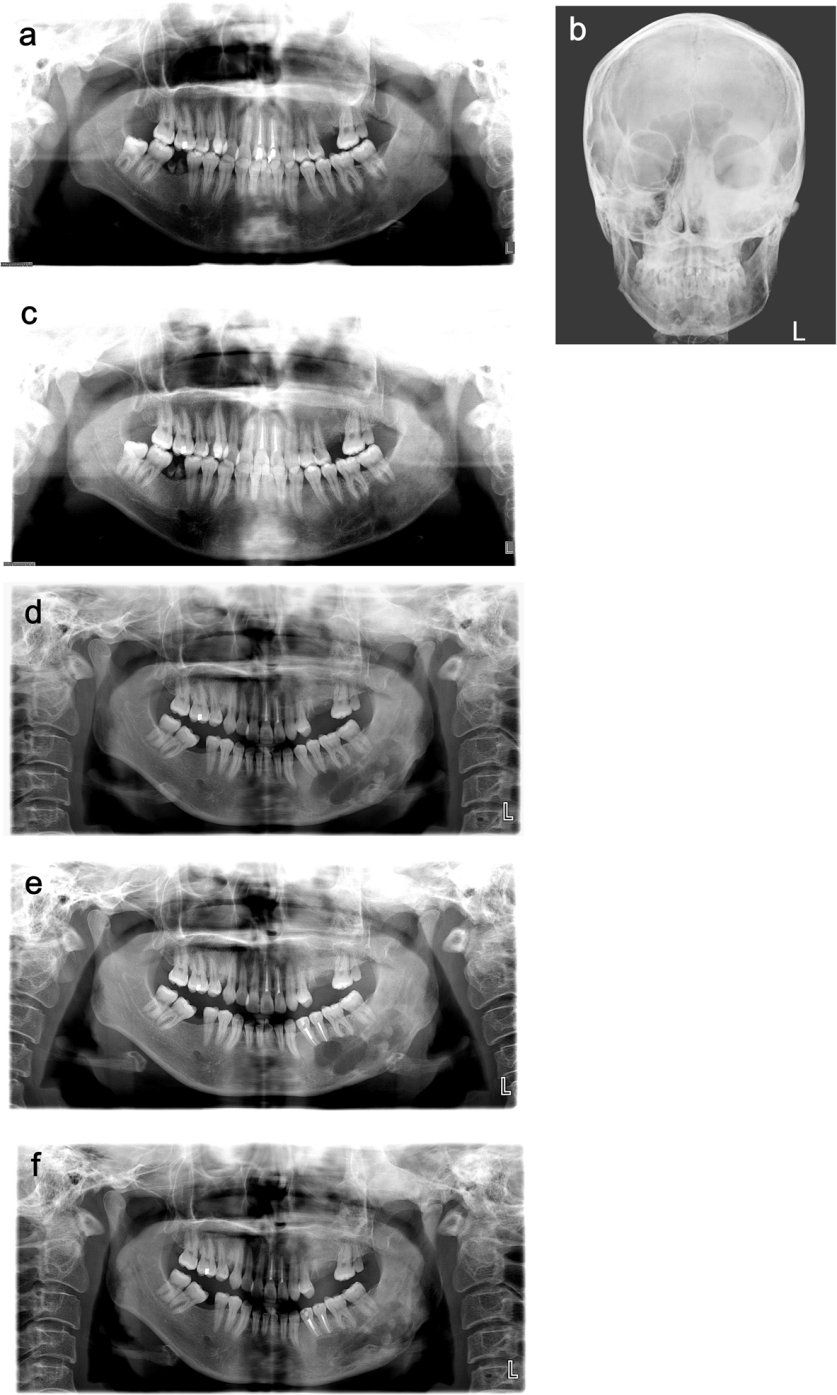
Radiographic images of the patient’s jaw and skull. **a** Panoramic view of the jaw at baseline taken at the first visit to the out-patient department (2007.5). **b** Posteroanterior projection (PA) view of the skull at baseline taken at the first visit to the out-patient department (2007.5). **c** Postoperative panoramic view of the jaw taken 18 months post-surgery (2008.11). **d** Panoramic view of the jaw taken at the out-patient department (2018.11). **e** Panoramic view of the jaw after cyst enucleation of the left mandibular lesion. **f** Postoperative panoramic view of the jaw 6 months after the surgery in November 2018 (2019.5)

**Fig. 2:**
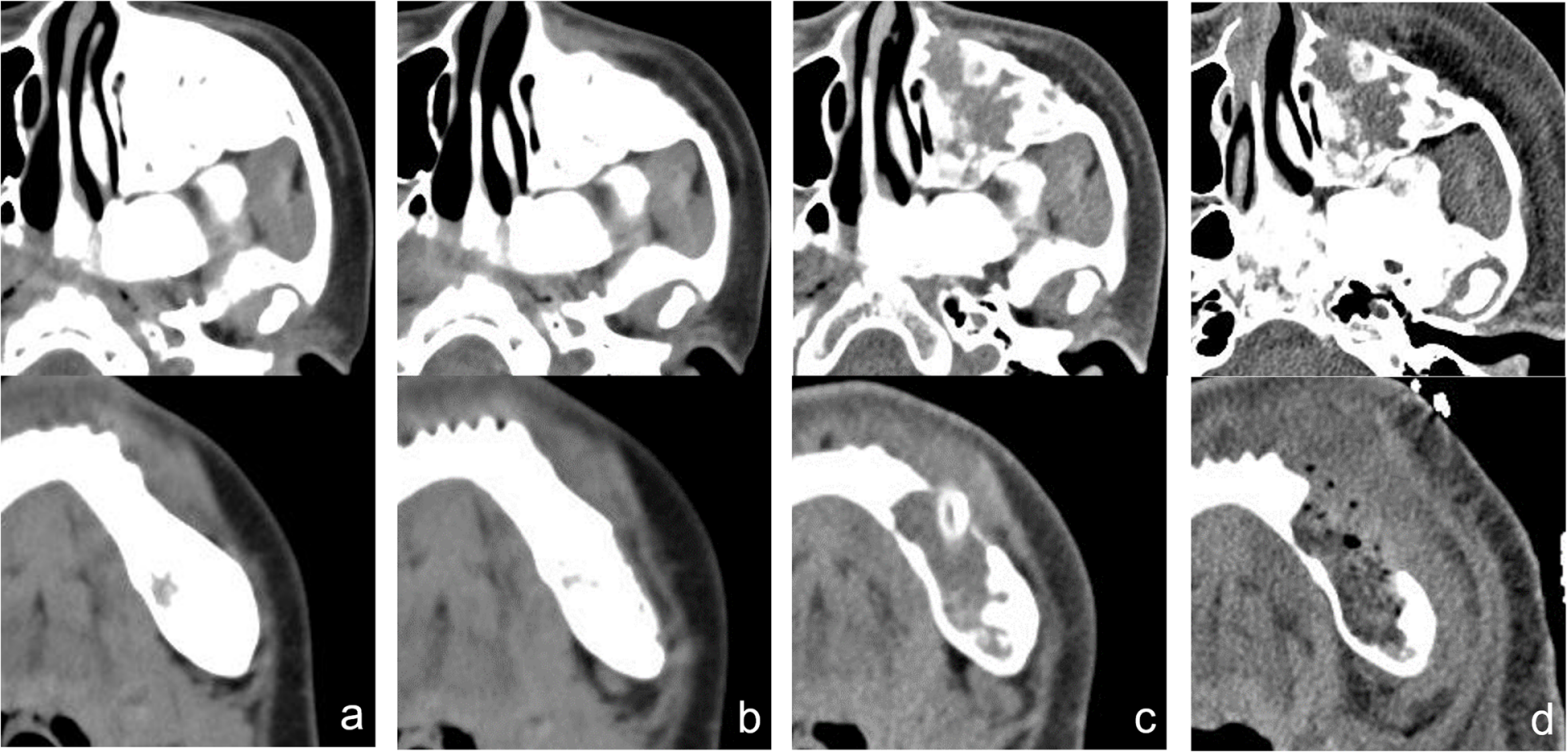
Contrast-enhanced facial computed tomography (CECT) images of the patient. **a** CECT image taken at the first visit to the out-patient department (2007.5). **b** CECT image taken 3 months postoperatively (2007.8). **c** CECT image after incisional biopsy and marsupialization (2018.11). **d** Postoperative CECT image after cyst enucleation on the left mandible in November 2018

In November 2018, a 30-year-old woman presented to the Department of Oral and Maxillofacial Surgery of Chonbuk National University Hospital with a complaint of pain and swelling in the left mandible that had appeared 10 days earlier. Eleven years ago, she was diagnosed with CFD (Fig. 1a, b) and had received bone contouring in the left zygomaticomaxillary complex and left mandibular region under general anesthesia in the same department (Fig. 2a, b). Postoperative healing was uneventful and 18 months postoperatively, there was no specific problem with the lesion (Fig. 1c). The patient had subsequently been lost to follow-up until November 2018.

A review of medical history prior to November 2018 confirmed that she had not received any dental treatment or suffered trauma to the painful left mandible area in recent months. Her pain intensity rating was 4 points on the numeric pain rating scale. Clinical examination revealed slight swelling in the left midface and left submandibular areas, along with bony expansion from the posterior of the left mandibular angle to the inferior aspect of the #34 tooth. The patient did not complain of hypoesthesia or pain when pressure was applied to the area. During an intraoral observation, the swelling was found from the distal aspect of #33 to the mesial vestibule area in relation to #36. During the endodontic examination, tooth mobility and percussion reactions were not observed in #34, 35, and 36. Moreover, the electric pulp test (EPT) showed normal response from #34, 35, and 36. No evidence of gum inflammation, such as periodontal pockets or gingival sulcus swelling and bleeding, was found during the periodontal examination. Furthermore, in the panoramic view, the dental origin with the possible infection source was not observed (Fig. 1d).

A well-defined multilocular radiolucent lesion in the left posterior mandibular region was identified on the panoramic radiograph, and the location of the lesion overlapped with the existing FD. In addition, amorphous calcified foci were observed inside the lesion (Fig. 1d).

**Fig. 3:**
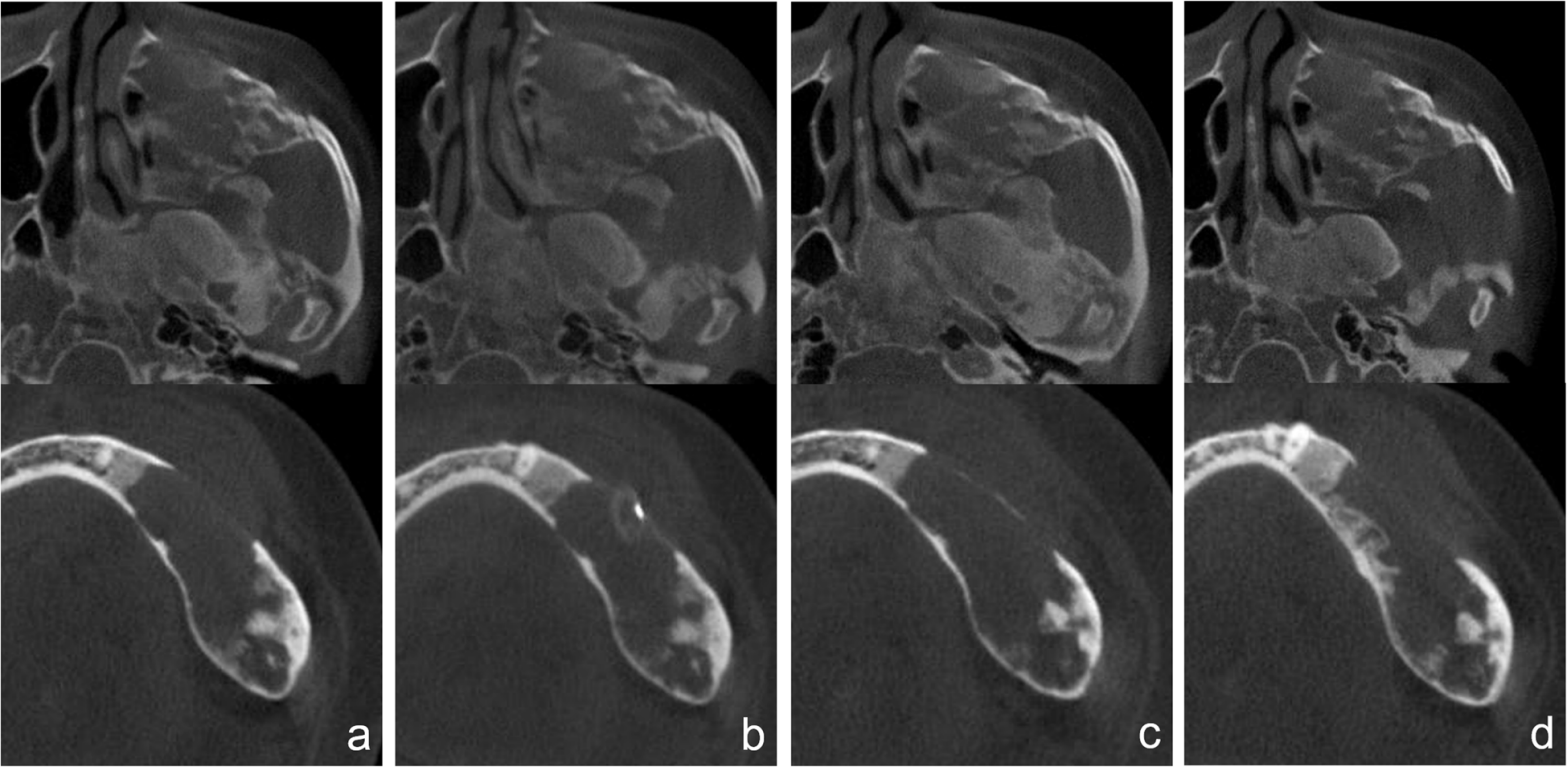
Cone-beam computed tomography (CBCT) images of the patient. **a** CBCT image taken at the out-patient department in November 2018. **b** CBCT image 3 weeks after marsupialization on the left mandible. **c** Postoperative CBCT image after cyst enucleation on the left mandible in November 2018. **d** CBCT image taken 6 months after the surgery in November 2018 (2019.5)

Cone-beam computed tomography (CBCT) showed an expansive bone lesion with a ground-glass appearance spanning the left frontotemporal bone, crista galli, orbital wall, ethmoid bone, sphenoid bone, zygoma, pterygoid plate, and maxilla regions (Fig. 3a). An ill-defined (partially well-defined) irregular osteolytic lesion was observed inside the left mandibular lesion, and cortical thinning, buccolingual expansion, and cortical destruction were also identified (Fig. 3a).

A decision to perform a marsupialization procedure was made to first control edema and pain, and second to take a biopsy. The marsupialization procedure was performed after an intraoral incisional biopsy of the area surrounding the #34 and #35 teeth, followed by root canal treatments on these teeth. The biopsy results revealed some evidence of chronic inflammation and that the lesion may be a bony lesion with inflammatory reaction rather than FD. During a 3-week observational period, the size of the lesion was unchanged according to clinical and radiological findings (Fig. 3b). Eventually, we decided that cyst enucleation under general anesthesia should be performed in the mandibular region. However, in the maxilla region, since there was no pain or discomfort, we decided to follow-up without any surgical treatment.

### Surgical note

**Fig. 4:**
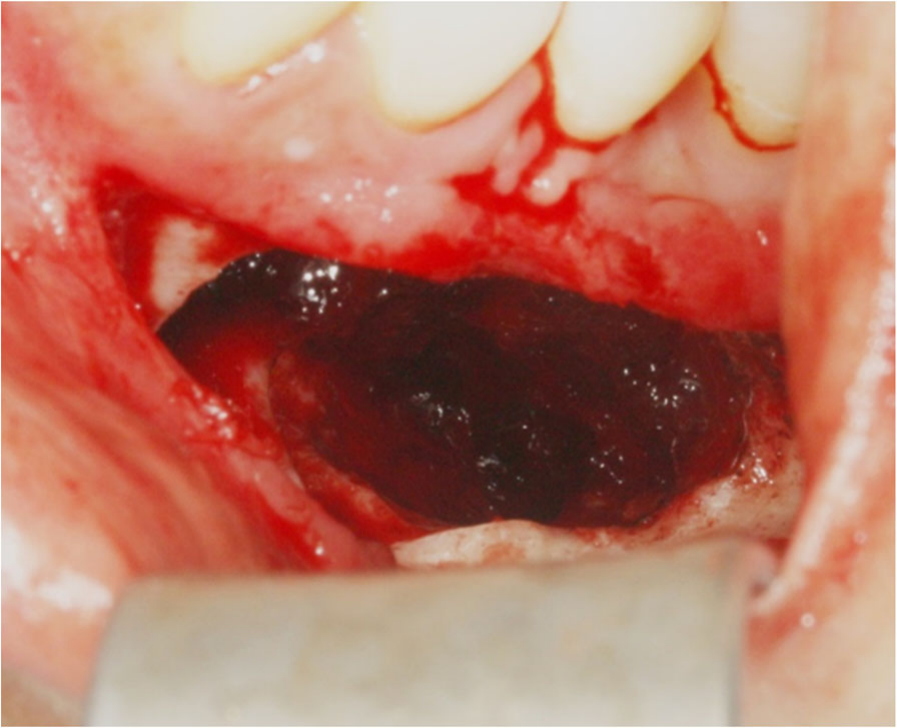
Intraoperative photograph of the left mandibular lesion after cyst enucleation

At the time of surgery, the lesion had expanded from the inferior aspect of the #34 tooth to the mesial root of the #36 tooth, with fibrotic tissue scattered within the lesion. Subsequently, the soft tissue lesion was removed by cyst enucleation. The perilesional bone and the roots of the #34, 35 teeth, and the mesial root of the #36 tooth were ground. Electrocautery was applied to the interior of the lesion and a thorough curettage was performed (Fig. 4).

### Pathological note

**Fig. 5:**
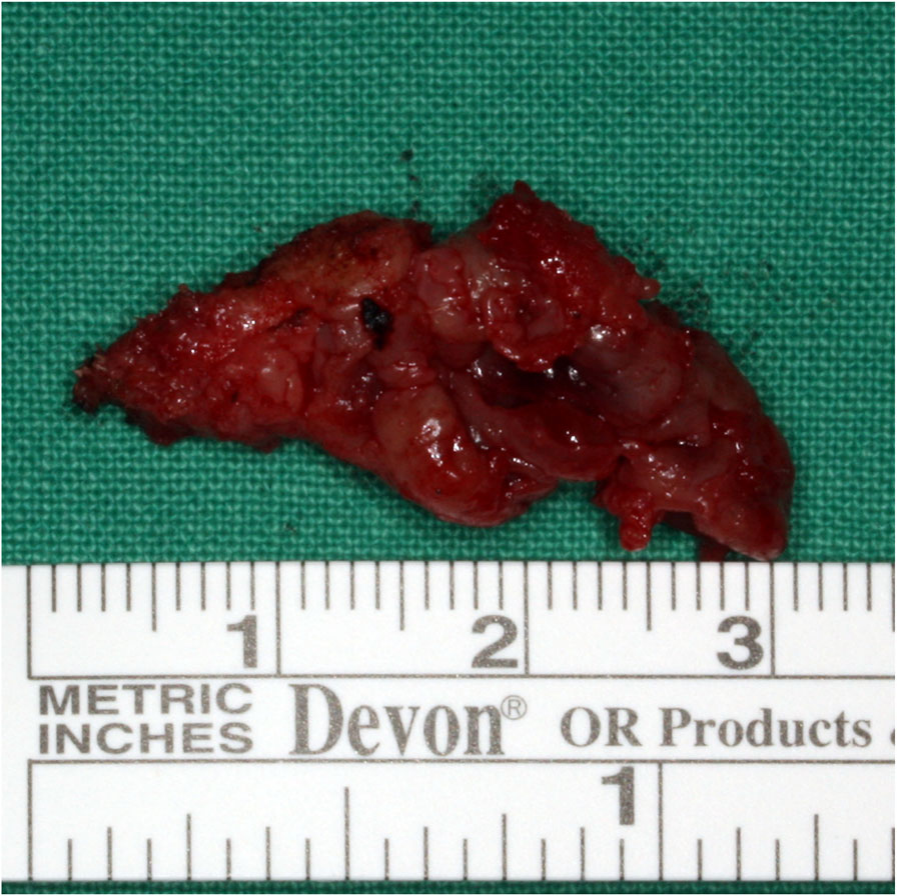
Photograph of the main mass

The lesions removed by cyst enucleation were sent for tissue biopsy. The largest lesion was approximately 3 × 2 × 1.5 cm and was lined by a thick, fibrotic tissue layer (Fig. 5).

**Fig. 6:**
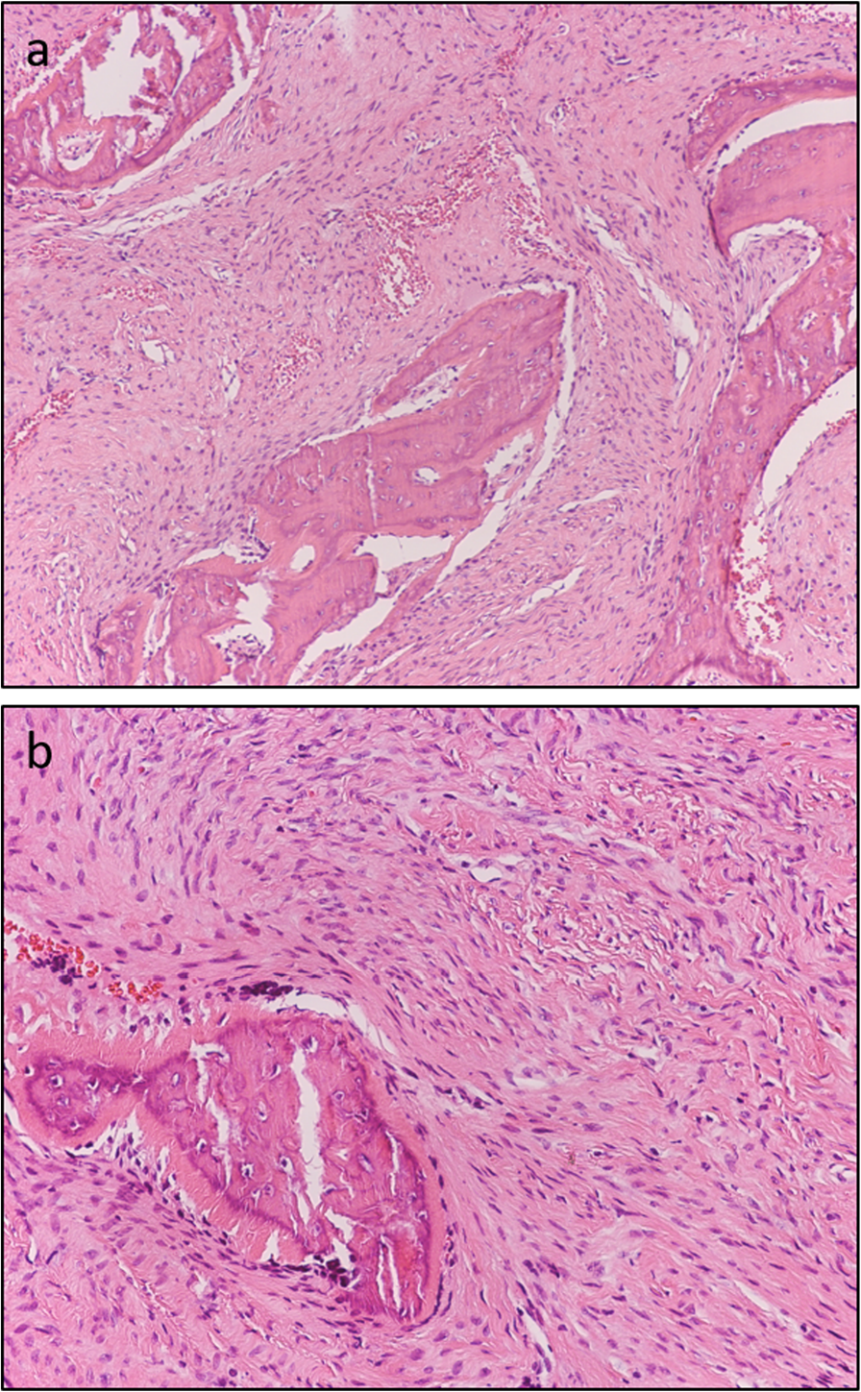
Histologic features of the mass. **a** Dense collagenous tissue surrounds the osseous trabeculae and peritrabecular clefting is present (H and E stain, original magnification: × 100). **b** Mitosis or atypia is not seen (H and E stain, original magnification: × 200)

Hematoxylin and eosin (H and E) stained sections showed dense collagenous tissue surrounding the osseous trabeculae, and peritrabecular clefting was present (Fig. 6a). Mitosis or atypia was not seen (Fig. 6b). The biopsy result revealed active nonspecific chronic inflammation with fibrosis.

In view of the radiographical and intraoperative findings, the absence of any history of trauma, a low probability of dental infection being the cause (as per endodontic and periodontal examinations), absence of evidence of malignant transformation (confirmed by histological findings) [13–15], the researchers confirmed that the case involved non-specific cystic degeneration in the CFD site. Postoperative healing was uneventful (Figs. 1e and 3c), as was the postsurgical follow-up over a 6-month period (Figs. 1f and 3d). Thereafter, the patient was lost to follow-up.

## Discussion

The FD cysts with nonepithelial lining have different histological characteristics, in which some are aneurysmal bone cysts, whereas others are simple bone cysts or nonspecific cyst degenerations [6]. The former two entities are characterized by cavities in bones filled with blood and lined by a layer of thick fibrous tissue [8]. Aneurysmal and simple bone cysts are sometimes considered as secondary phenomena of many benign and malignant bone tumors and tumor-like lesions. On the other hand, a lesion that does not have histological features of either an aneurysmal or a simple bone cyst is regarded as nonspecific cyst degeneration [8].

Cystic degeneration of CFD is most often found in the sphenoid and frontal bones of patients with FD. Pressure from the cysts on the optic nerves can cause acute optic nerve compression in patients with acute cystic degeneration (ACD) of CFD [16]. Cysts tend to show a more aggressive course, which may be due to their association with several potential mechanisms, rather than being determined by a single pathogenetic event. Some cysts are thought to be caused by disruption of venous diploic channels, while it has also been suggested that bone cysts may occur due to an intraosseous vascular defect causing intramedullary hemorrhage [17]. Cysts may expand rapidly depending on the site of onset, and can present with a variety of symptoms [8]. Sudden expansion due to the development of cystic degeneration may lead to sudden deterioration of vision [17]. The patient in our case report also presented with cystic degeneration of FD in the maxilla, but did not complain of vision deterioration.

According to the National Institutes of Health cohort study (unpublished observations), cystic degeneration occurred in approximately 5% of all patients with FD [17], whereas Bahk et al. reported that it occurred in approximately 8% of all patients [18]. Ferretti et al. estimated that cystic degeneration of FD covers the spectrum between a simple bone cyst and an aneurysmal bone cyst [6]. The important clinical feature of cystic degeneration of FD is a rapid increase in cyst size. This may be misdiagnosed clinically as an aneurysmal bone cyst, a simple cyst, or even a sarcomatous change of a pre-existing benign bony tumor. Therefore, a high index of suspicion is needed when there is a rapid increase in cyst size, as it may be caused by cystic degeneration in patients with FD [19]. Cystic areas within the involved bone are depicted on computed tomography (CT) scans as hypointensity. Therefore, CT scans should be obtained regularly every year until the lesion is stabilized [17]. Radiation therapy is not recommended for FD cysts due to a high potential for malignant transformation (up to 44%) [20].

Cystic degeneration of FD may exhibit a rapid increase in size, which could be misdiagnosed as a malignant transformation. If FD shows a clear lytic appearance or a rapid increase in size, the possibility of cystic degeneration or malignant transformation should be considered. Cystic degeneration of FD that shows an aggressive pattern on radiological findings tends to have poorly defined borders, osteolytic changes, and erosion of the cortex with periosteal reaction. This pattern is similar to that of the malignant transformation of FD [21], making it difficult to differentiate between cystic degeneration and malignant transformation of FD based on radiological findings alone [22]. To avoid unnecessary surgery, a preoperative biopsy should always be conducted [23].

Surgical treatment for FD is limited to cases involving esthetic or functional problems. It is recommended when FD poses a threat to important anatomical structures, such as the eyes or the optic nerves, causes significant esthetic deformities, or if severe pain is clearly associated with the FD process [24]. In some cases, cosmetic trimming of excess bone may be required [11, 25]. Some studies that have reported on surgical treatments for cystic degeneration of CFD are presented in Table 1.

**Table 1:** Studies that have reported on surgical treatments for cystic degeneration of craniofacial fibrous dysplasia (CFD)

Author	Onset age (years), sex	Location of cystic degeneration of CFD	Symptom	Surgical treatment	Pathology
Ferretti et al. [6]	12, M	Right mandible	Swelling	Enucleation	Benign fibro-osseous lesion
Muraoka et al. [7]	25, F	Left maxillary sinus	Swelling	Decompression	Fibrous dysplasia
Diah et al. [8]	12, M	Right frontal, Sphenoid, Occipital bone	Swelling	Resection	Aneurysmal bone cyst
Diah et al. [8]	22, F	Right sphenoid	Pain, swelling, visual deterioration	Resection	Walled, with chronic inflammation and hemorrhage
Nadaf et al. [9]	40, F	Both mandible	Swelling	Resection	Fibro-osseous lesion
Oostenbroek-Bisschop et al. [10]	40, F	Right mandibular condyle	Pain	Resection	Fibrous dysplasia with cystic degeneration
Saxena et al. [11]	9, M	Left ethmoid air cells area	Swelling, left nasal blockage and bleeding	Resection	Fibrous dysplasia with hemorrhagic cystic change
Holl et al. [12]	16, F	Left sphenoid	Visual deterioration	Decompression	Aneurysmal bone cyst
Bowers et al. [26]	24, F	Sphenoid	Visual deterioration	Decompression	Data does not exist

In this case, our patient had a rare case of cystic degeneration simultaneously in the maxillary and mandibular FD. The mandibular lesion showed discomfort and pain, and cyst enucleation was performed. However, follow-up was decided on the maxillary lesions because of the absence of pain or discomfort in that region. We found no noticeable differences in maxillary lesions on CBCT at 6 months follow-up (Fig. 3d). The postoperative site in the left mandible showed progressive bony healing without evidence of recurrence or an increase in lesion size (Figs. 1f and 3d). However, this was a short-term follow-up. Continuous follow-up is required for judgment of prognosis in the long term.

## Conclusions

Cystic degeneration in CFD cases is rare; cystic degeneration appearing in both the maxilla and mandible is even rarer. In this case, our patient had a rare case of nonspecific cystic degeneration simultaneously in the maxillary and mandibular FD. The mandibular lesion caused discomfort and pain, and cyst enucleation was performed. Continuous follow-up is required for long-term prediction of prognosis.

## Data Availability

Not applicable

## Abbreviations

ACD: Acute cystic degeneration
CBCT: Cone beam computed tomography
CECT: Contrast-enhanced computed tomography
CFD: Craniofacial fibrous dysplasia
CT: Computed tomography
EPT: Electric pulp test
FD: Fibrous dysplasia
MAS: McCune–Albright syndrome
